# Which return regime induces overconfidence behavior? Artificial intelligence and a nonlinear approach

**DOI:** 10.1186/s40854-022-00446-2

**Published:** 2023-01-18

**Authors:** Esra Alp Coşkun, Hakan Kahyaoglu, Chi Keung Marco Lau

**Affiliations:** 1 Department of Accountancy, Finance, and Economics, University of Huddersfield, Queensgate, Huddersfield, HD1 3DH UK; 2 Present Address: Department of Economics, Middle East Technical University, Ankara, Turkey; 3 Department of Economics, Dokuz Eylul University, Dokuzcesmeler Yerleskesi Buca-Izmir, Izmir, Turkey; 4 Business School, Teesside University, Middlesbrough, England, UK

**Keywords:** Overconfidence, Nonlinear Granger causality, Artificial intelligence, Feed-forward neural networks, Nonlinear impulse-response functions, Local projections, Return regime, G41, G15, C45

## Abstract

Overconfidence behavior, one form of positive illusion, has drawn considerable attention throughout history because it is viewed as the main reason for many crises. Investors’ overconfidence, which can be observed as overtrading following positive returns, may lead to inefficiencies in stock markets. To the best of our knowledge, this is the first study to examine the presence of investor overconfidence by employing an artificial intelligence technique and a nonlinear approach to impulse responses to analyze the impact of different return regimes on the overconfidence attitude. We examine whether investors in an emerging stock market (Borsa Istanbul) exhibit overconfidence behavior using a feed-forward, neural network, nonlinear Granger causality test and nonlinear impulse-response functions based on local projections. These are the first applications in the relevant literature due to the novelty of these models in forecasting high-dimensional, multivariate time series. The results obtained from distinguishing between the different market regimes to analyze the responses of trading volume to return shocks contradict those in the literature, which is the key contribution of the study. The empirical findings imply that overconfidence behavior exhibits asymmetries in different return regimes and is persistent during the 20-day forecasting horizon. Overconfidence is more persistent in the low- than in the high-return regime. In the negative interest-rate period, a high-return regime induces overconfidence behavior, whereas in the positive interest-rate period, a low-return regime induces overconfidence behavior. Based on the empirical findings, investors should be aware that portfolio gains may result in losses depending on aggressive and excessive trading strategies, particularly in low-return regimes.

## Introduction

*“Ignorance more frequently begets confidence than does knowledge: it is those who know little, and not those who know much, who so positively assert that this or that problem will never be solved by science” (Darwin, 1871).*

Charles Darwin

An individual’s level of self-confidence specifies their behavior, whether rational or irrational. Being overconfident depends on an individual’s circumstances and personal factors; therefore, it may arise from various motivations. Moore and Healy (2008) suggested three types of overconfidence: overestimation of one’s abilities (overestimation); when one believes that they are better than others (overplacement); and excessive certainty regarding the accuracy of one’s belief (overprecision). Meanwhile, Piehlmaier (2022) drew attention to the similarity of overconfidence to other behavioral biases, such as hindsight and self-serving biases, cognitive dissonance, and the illusion of control (Malmendier and Taylor 2015; cited in Piehlmaier 2022). Generally observed in different forms, overconfidence behavior originates from human evolution. Martin Seligman’s original works (2004, 2014) and psychological experiments clarified that our language, memory, and thoughts were systematically biased in a positive direction. The evolutionary biologist, Robert Trivers (2000), argues that self-deception has an ancient origin in the evolution of the brain. Wrangham (1999) proposed that natural selection favored positive illusions as they were likely to be advantageous for hunter-gatherer societies in warfare conditions, interpersonal combat, one-on-one disputes, bargaining, attracting allies, and deterring rivals. Moreover, it had critical consequences for survival, access to materials and resources, and reproductive opportunities. In investors’ case, attributing success to themselves, believing themselves to be better than others, or other reasons may be their motivation; however, the outcome of overconfidence is mostly observed in “overtrading,” when higher returns are targeted, according to the empirical literature. Therefore, causality between returns and trading volume has been accepted as evidence of overconfidence in the empirical literature. Based on the above discussion, overconfidence may sometimes be advantageous, and the experience of favorable outcomes due to overconfidence, such as positive returns following overtrading, may lead to a self-feeding process and hence persistence. Accordingly, overconfidence may be seen as rational on its merits, depending on the circumstances. Thus, it is essential to investigate the circumstances under which overconfidence may be designated as advantageous.

The analysis of overconfidence in stock markets was first conducted by Barber and Odean (2000, 2001), Daniel et al. (1998, 2001), and Odean (1998a, b, 1999). The number of studies focusing on developed (Berg and Rietz 2019; Deaves et al. 2010), emerging (Phan et al. 2020; Qasim et al. 2019), and both (Abbes 2013) stock markets has increased in recent years. The question of overconfidence behavior in emerging stock markets has received considerable attention because empirical studies have demonstrated that market anomalies tend to be more dominant in emerging markets (Abdeldayem and Mahmoud 2013; Chen et al. 2007; Metwally and Darwish 2015; Wang 2008).

We investigate the existence of overconfidence in an emerging market, Borsa Istanbul (BIST), which is a remarkable case to be examined because Turkey experienced economic and financial crises in the period 2018–2019, including a currency crash, a diplomatic crisis with the United States (U.S.), and a Covid-19-based economic downturn (Akçay and Güngen 2018, 2019; Arbaa and Varon 2019; Oyvat 2018; Sumer and Ozorhon 2020). Interestingly, during this period of turmoil, the number of active investors in BIST increased by 65% from 12.31.2019 to 01.15.2021 (Central Registry Agency (CRA) 2020). From 2015, the starting date for our data, to 2021, the number of active investors almost doubled, rising from 1,064,754 in 2015 to 2,002,873 in 2021 (CRA 2014, 2020). Meanwhile, based on the real rate of return on the BIST100 (Borsa Istanbul) during the period 2015–2020 (see Appendix B, Fig. 7), it is interesting that most investments returned a negative real rate in all periods except 2017. Particularly during the economic crisis period (2018–2019), investors earned negative real yields; despite this, there was a large increase in the number of active investors during the period, which indicates that behavioral motivations may have played a role in this emerging market. Motivated by the above theoretical frameworks and market facts, we investigated causality between returns and trading volume to test for the presence of overconfidence behavior in BIST, following several authors (Barber and Odean 2000, 2001; Daniel et al. 1998, 2001; Odean 1998b) who previously suggested this framework.

Despite the great importance of overconfidence behavior, which is seen as the most plausible reason for many disasters, crises, and bubbles (Akerlof and Shiller 2010; Johnson 2004; Johnson and Fowler 2011; Johnson and Levin 2009; Johnson and Tierney 2011; Tuchman 2011), what remains unclear is the impact of market states on overconfidence behavior. One question that must be asked is whether return-regime type influences the overconfidence attitude. Investors’ psychology is expected to be influenced by low-/high-return regimes (Burnside et al. 2011; Daniel et al. 2001; Gervais and Odean 2001; Kim and Nofsinger 2007; Meier and De Mello 2020; Shiller 2000); therefore, overconfident investors may behave differently in different return regimes. There has been little quantitative analysis of the impact of market conditions on investors’ overconfidence. Some studies have investigated bear and bull market conditions (Chuang and Lee 2006; Kim and Nofsinger 2007; Shi and Wang 2013) and different return regimes (Liu et al. 2016), while others have explored different research questions regarding overconfidence by considering market conditions (Jlassi et al. 2014; Namouri et al. 2018). However, until recently, there have been no reliable studies that have examined how overconfidence behavior varies in low-/high-return regimes by forecasting changes in the investment horizon in daily increments. Our methodology in this study allows us to observe changes in trading volume in both low- and high-return regimes. The positive/negative responses of overconfidence behavior to changes in return and magnitude of the responses highlight the dynamic, daily reaction of the behavior. Alternative methodologies may yield results of a positive/negative relationship; however, a nonlinear impulse-response analysis presents a dynamic forecast in low-/high-return regimes for a specific time horizon. Thus, it separately highlights all impulses and responses in low- and high-return regimes for a specific duration, which is essential to ascertaining a more powerful policy for policymakers and strategies for investors. Moreover, we examined overconfidence behavior for subperiods based on positive/negative interest rates to better understand the motivation for overconfidence.

The empirical contribution of this study is twofold: First, to the best of our knowledge, this study is the first to attempt to investigate the presence of overconfidence bias using an artificial intelligence (AI) method. Second, we examine the variation in overconfidence behavior in different return regimes, which may provide rich insights for formulating practical suggestions for investors, portfolio managers, and policy makers. Therefore, we apply the feed-forward, neural-network (FFNN), nonlinear, Granger causality test and nonlinear impulse-response functions based on local projections (LPIRFs) (Jordà 2005). Analyzing the different return regimes provides rich outcomes, which is the primary contribution of the study. However, the main contribution of the study is empirically driven. Additionally, we attempt to review the roots of overconfidence bias to better explain the underlying reasons for this irrational behavior, which suggests more realistic policy implications.

This paper is divided into five main sections. The second section both theoretically and empirically reviews the relevant literature on overconfidence behavior. The third section explains the data structure and methodology applied in the study. In the fourth section, the empirical results are reported and discussed. The last section concludes with a summary of the results, recommendations for investors, and some policy implications.

## Literature review

### The historical roots of overconfidence

The overconfidence phenomenon has its roots in “The Wealth of Nations,” by Adam Smith (1776), which provided a discussion on a man’s overestimation of himself in a variety of situations. Smith believed that overconfidence was a universal sense, and referred to it as an “ancient evil:”

“The overweening conceit which the greater part of men have of their own abilities is an ancient evil remarked by the philosophers and moralists of all ages. Their absurd presumption in their own good fortune has been less taken notice of. It is, however, if possible, still more universal. There is no man living who, when in tolerable health and spirits, has not some share of it. The chance of gain is by every man more or less overvalued, and the chance of loss is by most men undervalued, and by scarce any man, who is in tolerable health and spirits, valued more than it is worth.”

Keynes (1936) also mentioned “animal spirits” and “optimism,” and emphasized that everything depended on waves of irrational psychology, in his “General Theory of Employment, Interest and Money.” Keynes brought into focus the fact that rationality was impossible under uncertainty due to the absence of perfect knowledge or calculable probabilities (Dow 2011). Keynes (1936) thought that in addition to the instability caused by speculation, there was the instability caused by the fact that a large proportion of our positive behaviors, whether moral, hedonistic, or economic, relied on spontaneous optimism rather than a quantitative expectation. Akerlof and Shiller (2010) claimed that overconfidence was the most prominent bias that relied on a core of volatile beliefs.

Kahneman and Tversky (1979a, b) suggested overconfidence bias and the factors that contributed to overconfidence among investors, and introduced “Prospect Theory” to provide explanations for violations of the expected utility theory. Following Kahneman and Tversky (1979a, b), several studies attempted to suggest new models to better explain the irrational nature of financial markets. For instance, Scheinkman and Xiong (2003) proposed a model to describe the bubbles generated from speculative trading among agents with heterogeneous beliefs that arose from their overconfidence. Daniel et al. (2001) suggested a theory of asset pricing and argued that biased learning could cause traders, based on experience, to become more overconfident instead of converging toward rationality, and that overconfident investors could earn higher expected profits than rational traders.

Roll (1986) was the first author to introduce the optimism and overconfidence approach to corporate finance, with his “hubris” theory. Roll’s hubris theory suggested that managers/chief executive officers (CEOs) of acquiring firms made valuation errors and were therefore too optimistic about potential synergies in proposed takeovers. Consequently, they overbid target firms to the detriment of their stockholders (Brown and Sarma 2007). Camerer and Lovallo (1999) directly tested the idea that overconfidence caused business-entry mistakes by simultaneously measuring economic decisions and personal overconfidence. Malmendier and Tate (2005) found that overconfident CEOs’ investments were significantly more responsive to cash flows, particularly in equity-dependent firms.

The term “overconfidence” has been widely used in psychology, starting from the 1960s (Skała 2008). “Overconfidence,” as a form of miscalibration, is usually applied by economists in studies of overconfidence in the context of positive illusions, i.e., the better-than-average effect (overplacement), illusion of control, and unrealistic optimism (Skała 2008). Taylor and Brown (1994) claimed that most people exhibited positive illusions/overconfidence in three important domains: “i) They view themselves in unrealistically positive terms. ii) They believe they have greater control over environmental events than is the case. iii) They hold views of the future that are rosier than base-rate data can justify.” Consequently, overconfidence leads to an error in judgement or decision making, which in turn leads to overestimating one’s capabilities and/or underestimating an opponent, the difficulty of a task, or possible risks (Johnson and Fowler 2011).

The advantages and disadvantages of overconfidence are discussed as two sides of the same coin in the relevant literature. For instance, Taylor and Brown (1994) have found that positive illusions lead to “higher motivation, greater persistence, more effective performance, and ultimately greater success.” Daniel and Titman (1999) argued that overconfidence was a trait that likely arose through evolutionary selection. They believed that a trait such as overconfidence would be eliminated through natural selection if it simply resulted in irrational decisions. However, because overconfidence improves genetic survival in other ways, experimental evidence in its support is even stronger.

However, Johnson (2004) emphasizes that “too much” confidence may lead to a disaster and losses. As Moore and Healy (2008) claim, overconfidence is offered as an explanation for wars, strikes, litigation, entrepreneurial failures, and stock market bubbles (Johnson 2004; Malmendier and Tate 2005; Neale and Bazerman 1985; Odean 1999).

In light of the above discussion, both the existence of and level of overconfidence are paramount and prominent factors in determining whether the outcomes of a given behavior are advantageous or disadvantageous. Therefore, it is crucial to thoroughly investigate the level of overconfidence and outcomes of investors’ behavior. If overconfidence is observed, for instance, in a high-return regime, resulting in negative returns, it can be considered as irrational behavior. However, overconfidence in a low-return regime with positive returns may be considered rational behavior on its merits. The motivation in such a period, for instance, may increase the real rate of return if real interest rates are negative. From this viewpoint, we investigated the level of overconfidence in low- and high-return regimes and for subperiods based on positive/negative interest rates to better observe the motivation for overconfidence and whether its outcomes were profitable.

### Empirical studies

Price bubbles can arise in markets and lead to financial crises when irrational prices related to overconfidence are greater than effective price levels. Therefore, it is critical to investigate factors in investors' irrational decisions, such as overconfidence. Numerous studies in the literature have investigated whether overconfidence behavior exists among decision-making units in both advanced and emerging financial markets.

#### Emerging stock markets, including BIST

Many studies have attempted to examine whether investors exhibit overconfidence behavior in emerging stock markets. Using data on 46,969 individual and 212 institutional investor accounts trading on the Shanghai and Shenzhen stock exchanges, Chen et al. (2007) tested for the presence of overconfidence behavior in the emerging market for the period May 20, 1998 to September 30, 2002. The results revealed that while there was a higher rate among individual investors, there was also overconfidence among institutional investors. Chen et al. (2007) also emphasized the importance of testing for behavioral biases in emerging markets because cultural or educational systems might lead to differences in investors’ behavior. Kansal and Singh (2018) surveyed investors living in the city of Delhi. The authors claimed that overconfidence behavior was associated with over-trading, but that there was no difference between the sexes in overconfidence behavior. Metawa et al. (2019) surveyed 384 local, foreign, institutional, and individual investors living in Egypt. The results showed that investors' intuition, overconfidence, age, gender, and level of education positively impacted investment decisions. Arifin and Soleha (2019) surveyed students of the faculty of economics at the Islamic University of Indonesia who traded on the stock market, mostly in Yogyakarta. The authors, who claimed that risk-seeking investors were dominant in the stock market, which meant that overconfidence was prevalent, pointed out that overconfidence was related to investors' attitude toward risk, while financial literacy did not affect overconfidence. Qasim et al. (2019) surveyed 150 subjects who invested in the stock market in Pakistan. In the study, it was found that investors were overconfident. Zhang et al. (2019) used data on 16,615,047 transactions performed by 179,049 households who invested in the Chinese stock market during the period October 2014 to April 2015 to analyze overconfidence behavior. The results of the study revealed that investors were overconfident. Ngene and Mungai (2022) examined the asymmetric and intertemporal causal relationship between the stock returns, trading volume, and volatility of eight African stock markets. Adopting a nonlinear approach, the authors found evidence of overconfidence bias based on a quantile regression model.

Several researchers have used both time series analysis and experimental research or survey techniques in BIST to investigate the validity of overconfidence behavior. Korkmaz and Çevi̇k (2007) examined overconfidence behavior in BIST using the closing prices and trading volumes of 114 companies’ stocks between May 1995 and October 2006. According to the results, overconfident investors traded more after a market gain and became more active in a winning/bull market. Moreover, there was insufficient evidence to confirm that overconfident investors traded more in risky assets after market gains. Kapucu and Emektar (2009) used transaction data to investigate the heuristics that affected investment decisions for individual investors in the service sector who invested in equity through a brokerage house in Istanbul province, Anatolian Side. The authors found evidence that the disposition effect, representativeness heuristic, framing and reflection effects, overconfidence, and heuristics were effective in investors’ stock investment decisions, whereas the certainty effect was not significant. Bolaman and Yücel (2012) tested two hypotheses: “return increases overconfidence behavior” and “overconfidence increases market volatility” in BIST for the period April 1991 to January 2011. Based on the results, they found positive causality from return to trading volume, while there was no significant relationship between overconfidence and volatility. Tekçe and Yilmaz (2015), using investment transaction data from the CRA (Turkey) for 2011, found that individual stock market investors exhibited overconfidence behavior.

Many studies have conducted surveys to analyze overconfidence behavior in BIST. Asoy and Saldanlı (2017) surveyed 423 individual investors to analyze the overconfidence and over-optimization behaviors of investors trading in BIST. The results showed that individual investors relied on personal intuition and analysis, were optimistic in their expectations for the future and, according to the results of multilinear regression analysis, gender, age, sector experience, and monthly income were descriptive variables of cognitive biases. Çetiner et al. (2019), who surveyed 115 participants, investigated individual investors’ attitudes. According to the results of the study, the participants were more confident based on their answers to the "overconfidence" question when it was directly asked than when it was indirectly asked. Tekin (2020) surveyed a total of 522 students studying at different universities and analyzed the validity of overconfidence, control illusion, and over-optimism biases. In the study, the author suggested a scale of behavioral bias that could be used in studies to examine the impacts of overconfidence, control illusion, and over-optimism biases on individuals’ financial decision-making processes. Overall, the results of empirical studies on the presence of overconfidence in BIST revealed that investors were overconfident, which is consistent with those of studies on other emerging stock markets.

#### Advanced stock markets

Overconfidence behavior has also been examined in advanced stock markets, while some studies have included a comparison of the stock markets in terms of overconfidence behavior. Deaves et al. (2010) tested for the existence of overconfidence using data from a monthly survey called ZEW Finanzmarkttest with 350 financial market participants in Germany. The results of the study implied that market experience was not effective in calibrating behaviors. While market participants were overconfident, this behavior was not associated with self-attribution bias. Glaser and Weber (2009) used a dataset comprising individual investors from a German online broker in the period from January 1997 to mid-April 2001. The results of the study suggested that high past portfolio returns induced investors’ overconfidence due to a self-attribution bias. However, the authors observed that high past market returns did not lead to more risk-taking. Using closing-price and trading-volume data for the stock market indexes of 15 countries and regions (U.S., Canada, Brazil, Mexico, France, United Kingdom, Switzerland, Australia, Hong Kong, Japan, Korea, Malaysia, Singapore, India, and Kuwait), Abbes (2013) examined whether overconfidence behavior was an explanatory factor in the 2008 global crisis. According to the findings, bad news impacted investors more than good news, while in all countries except Japan and Singapore, there was a positive and meaningful relationship between volatility and trading volume as an indicator of overconfidence. Phan et al. (2020) investigated the stock exchanges of Vietnam, Thailand, and Singapore for the period January 1, 2014 to December 31, 2018. Based on the results of the study, investors in Vietnam and Singapore were overconfident, while those on the Thai stock market were underconfident.

Regarding existing empirical studies on overconfidence bias and relying on several studies’ theoretical frameworks (Barber and Odean 2000, 2001; Daniel et al. 1998, 2001; Odean 1998b), we formulate the first hypothesis on the presence of overconfidence in BIST as follows:

##### Hypothesis 1

The current trading volume is positively related to past market returns.

#### Studies employing IRFs

Several studies have employed IRFs to analyze the responses of trading volume to market returns in a selected time horizon. However, these studies have employed linear impulse responses to analyze financial data, which are known to be nonlinear (see Song et al. 2021; Tank et al. 2021). None of these studies considered nonlinear impulse responses or any kind of impulse responses in BIST. Daniel et al. (1998) proposed a theory based on investor overconfidence and fluctuations in confidence as a result of the biased self-attribution of investment outcomes in their study. They used an IRF in addition to several methods. According to the theory, investors overreact to private-information signals and underreact to public-information signals. Statman et al. (2006) applied vector autoregression (VAR) and an IRF to test the overconfidence hypothesis using monthly data. The key findings of the study suggested that market-wide turnover and lagged market-wide returns were positively related, while overconfidence was valid in New York Stock Exchange/American Stock Exchange (NYSE/AMEX) non-fund common stocks. Chuang and Lee (2006) investigated overconfidence bias using IRFs by considering the response of stock price to private- and public-information shocks. They applied Granger causality analysis between return and trading volume to test for the presence of overconfidence. However, although they used different variables in the IRF analysis, their overall results provided evidence of overconfidence bias. They also investigated investors’ asymmetric trading behavior and found that investors did trade more aggressively in a bull market than in a bear market.^1^ This finding is consistent with Daniel et al.’s (2001) and Gervais and Odean’s (2001) discussions. Metwally and Darwish (2015) investigated whether investors trading on the stock exchange in Egypt were overconfident by applying VAR, delay selection, IRFs, and the Granger causality test for the period 2002–2012. They found that investors behaved overconfidently on the Egyptian stock exchange. Based on the IRF results, the response of trading volume to a shock to market return existed until the fifth lag. Moreover, in lag one, the response was not evident, while it turned large and positive in lag two. In the third lag, the impulse became negative and disappeared after the fifth lag. Alsabban and Alarfaj (2020) used monthly return and trading-volume data for the period 2007–2018 and employed VAR, Granger, and an IRF to test for the presence of overconfidence behavior in the Saudi stock market. The results indicated that investors were overconfident and, according to the IRFs, a one-standard deviation shock to market return in the first period was followed by a 0.4% increase in the 2nd month’s market turnover, implying that overconfidence was valid over the short horizon. Sheikh and Riaz (2012) analyzed the Karachi Stock Exchange in Pakistan using VAR and an IRF. The results implied that an impulse response of market return to a one-standard deviation shock to market turnover resulted in an approximate a 3% increase in the next month's return. The response was found to be statistically significant for up to three months. Prosad et al. (2017) applied an IRF to the Indian stock market. The results of the study revealed that the impact was highest for the first period (31%), declined in the second and third periods, and remained stable between 6 and 7%. Shrotryia and Kalra (2021) investigated 46 global stock markets using VAR and an IRF. Based on the results, the positive impulse responses were persistent over all 10 days for developed countries and regions (France, Hong Kong, Japan, Netherlands, South Korea, Sweden, and the U.S.), emerging countries and regions (Brazil, China, Egypt, Indonesia, Malaysia, Saudi Arabia, Taiwan, Thailand, and Turkey), and frontier countries (Jordan, Oman, Romania, and Vietnam) markets. However, the authors found that the responses were nonsignificant for Hong Kong region and Malaysia. Overall, the results of the above studies suggest that the response of trading volume to shocks to returns is high in the first period but decreases in time and disappears over the short time horizon.

Consistent with the above studies that employed IRFs to test whether the responses of trading volume to return shocks were persistent or temporary and found evidence for the former, we tested the following hypothesis:

##### Hypothesis 2

The responses of trading volume to return shocks are temporary in both regimes.

We tested Hypotheses 3 and 4 below, consistent with studies that investigated the impact of market states on overconfidence behavior. These studies found evidence of asymmetries across different market states such as up/down or bear/bull markets and stronger overconfidence behavior in up and bull markets (Chuang and Lee 2006; Daniel et al. 2001; Gervais and Odean 2001; Gupta et al. 2018; Jlassi et al. 2014; Kim and Nofsinger 2007; Liu et al. 2016; Namouri et al. 2018; Shi and Wang 2013):

##### Hypothesis 3

The responses of trading volume to return shocks exhibit asymmetries in different return regimes.

##### Hypothesis 4

The responses of trading volume to return shocks are greater in a high-return regime.

For the above hypotheses, there are no clear, a priori expectations based on theory; however, the null hypotheses represent the expectations based on the empirical literature. Our regime definition is different from those in studies that consider up/down or bear/bull market conditions; however, a high-return regime may be more related to a bull market than to a bear market (Kim and Nofsinger 2007). Therefore, Hypothesis 4 refers to related empirical studies that found evidence of stronger overconfidence in a bull market.

Empirical studies reveal that overconfidence is a prevalent phenomenon in financial markets both in advanced and in emerging stock markets. Previous studies that examine overconfidence behavior in BIST considering different periods and using several methodologies generally find evidence of overconfidence bias. However, to the best of our knowledge, our study is the first in the literature to apply an AI application and nonlinear IRFs to analyze overconfidence bias. To examine overconfidence behavior, we employed the FFNN, nonlinear Granger causality test as an AI application for the first time, relying on its novelty. Although several studies employed IRFs in an attempt to test for the presence of overconfidence in stock markets (e.g., Alsabban and Alarfaj 2020; Sheikh and Riaz 2012; Statman et al. 2006; Zaiane and Abaoub 2009), ours is the first to apply nonlinear LPIRFs, which allow for a distinction between market states as low- and high-return regimes to ascertain the responses of trading volume to return shocks in different regimes.

## Data and empirical methodology

In the study, we used the BIST100 index’s closing-price and trading-volume data in the daily frequency for the period 01.01.2015–05.12.2020. In the empirical literature, some studies use monthly data (e.g., Alsabban and Alarfaj 2020; Metwally and Darwish 2015; Sheikh and Riaz 2012; Statman et al. 2006) as several studies have claimed that changes in investors’ overconfidence occur over monthly or annual horizons (e.g., Gervais and Odean 2001; Odean 1998b, a; Statman et al. 2006). However, many studies have found evidence that overconfident investors frequently monitor their portfolios and generally very often review their investment strategies daily (Abreu and Mendes 2012; Strahilevitz et al. 2015; Tourani-Rad and Kirkby 2005). Moreover, Bajzik (2021) pointed out that the use of monthly data or VAR models makes the effect of trading volume on returns substantially more negative, thus suggesting caution. Therefore, we used daily data to observe overconfidence, following the relevant literature that suggests that daily activities are related to overconfidence. We obtained the data from the Bloomberg Terminal.

We applied two new methodologies: first, as an AI technique, the FFNN, nonlinear Granger causality test recommended by Montalto et al. (2015) and Calvo-Pardo et al. (2021); and second, as another nonlinear^2^ approach, nonlinear LPIRFs. To the best of our knowledge, our study is the first in the overconfidence-bias literature to apply the abovementioned AI application and LPIRFs to test for the presence of overconfidence behavior. The raw data and codes used in the study are publicly available on GitHub website via the link (https://github.com/behaviouralfinancier/overconfidenceAI).

Before we explain the novelty of the abovementioned approaches from a technical perspective, it is necessary to clarify how these techniques help advance research in finance, particularly behavioral finance. An investment process is often driven by many kinds of biases and heuristics that make the relationships between financial variables such as stock prices, trading volumes, returns, market caps, etc. nonlinear rather than linear. A nonlinear relationship means that an effect can create a cause that in turn can create an effect via feedback in the first system, resulting in loops. For instance, in our case, trading volume may increase after positive returns at time *t*, and this increase in trading volume may also increase or decrease the returns each time. At time *t* + 1, if returns increase/decrease, this may lead trading volume to increase/decrease based on the dominant behavior in the market. Daily, each time a return changes, the trading volume also changes; however, not only the direction but also the magnitudes of these changes may affect the sequential behavior. Moreover, the return regime in a market may also influence the reactions of trading volume to changes in return. Therefore, the relationship between trading volume and return shows nonlinearities and can be analyzed through nonlinear approaches. Therefore, FFNNs are used in our study as the first step because neural networks (NNs) rely on data training to learn and improve their accuracy over time. Using the FFNN, nonlinear Granger causality test, a return is used as an input layer while trading volume is used as the output layer, with one hidden layer in the model, thus allowing trading volume to feedback return and create a feedback loop to learn from the data. This means that each day’s different directions of changes in return (increase/decrease) and different magnitudes of changes have different impacts on the trading volume. By analyzing and classifying all responses of trading volume to return changes, the model determines a threshold in the background and yields a result of causality or non-causality. In the second step, nonlinear impulse-response analysis is used to distinguish between the responses of trading volume to return changes in low-/high-return regimes. Employing LPIRFs enables us to observe each response of trading volume to a change in return as well as the feedback of trading volume to return in different return regimes daily. The analysis illustrates how the magnitude and direction of a change in return affect trading volume on sequential days as well as how the changes in trading volume affect the returns. Consequently, by employing the abovementioned techniques, we can observe the dynamic overconfidence behavior; in other words, the daily change in overconfidence behavior can be obtained from the model outputs.

FFNNs are the most common statistical tools used to perform non-parametric regressions (Montalto et al. 2015). Han and Wang (2009) claim that the advantage of artificial neural networks (ANNs) is the ability to learn the mapping from the input to the output without a priori knowledge of the actual physical processes that describe the large scale of arbitrarily complex nonlinear problems. Moreover, ANN prediction uses all the values generated in the past.^3^

We estimated the LPIRFs instead of VAR or structural vector autoregressive (SVAR) models.^4^ Many studies in the literature have used the local projections (LP) method since Jordà (2005) recommended it (e.g., Adämmer 2019; Auerbach and Gorodnichenko 2013, a; Barnichon and Brownlees 2019; Favara and Imbs 2015; Hamilton 2011; Jordà et al. 2015, 2020; Jordà and Taylor 2016; Owyang et al. 2013; Swanson 2021; Tenreyro and Thwaites 2016). Ronayne (2011) claimed that LP techniques warranted inclusion in any thorough work on macro-econometric dynamics. Meanwhile, Kučinskas and Peters (2019) proposed a new framework for measuring biases such as overconfidence using LPs. According to the study, LPs are useful tools to address whether expectations exhibit non-linearities or state dependence.

The novelty of these two models can be explained as follows:

Advantages of FFNN, nonlinear Granger causality test:

i) ANN prediction uses all the values generated in the past and has several advantages. For instance, FFNNs can be trained using well-established optimization algorithms (Tacchino et al. 2020).
ii) Moreover, AI applications, particularly ANN models, perform better than conventional models, according to the related literature (Ellis and Wilson 2005; Tiwari et al. 2020; Zhong and Enke 2019; Zimmermann et al. 2001).
iii) Linear models lead to incorrect results because real-world systems show nonlinear dependencies between series (Tank et al. 2021). Nonlinear approaches commonly apply additional models to detect interaction in time series. With these additional models, additional nonlinear effects can be detected in the history of each time series.
iv) The FFNN has a basic structure and a wide range of applications, and can estimate any continuous and square-integral functions with arbitrary precision (Yang and Li 2017).
v) Unknown dependence structures between elements of high-dimensional, multivariate time series with weak and strong persistence can be accommodated using this method (Calvo-Pardo et al. 2021).

The LP model has many advantages over the traditional SVAR approach, including the following:

i) LP models are easier to estimate because they rely merely on simple linear regressions;
ii) t-point or joint-wise inference is easily conducted;
iii) the LP method is more robust to misspecifications, and does not suffer from the curse of dimensionality inherent in VARs;
iv) they easily accommodate experimentation with highly nonlinear and flexible specifications that may be impractical in a multivariate context (Jordà 2005) (also see Adämmer 2019; Barnichon and Brownlees 2019; Montes-Rojas 2019);
v) LPs are appealing because they are flexible and enable nonlinear and state-dependent impulse responses that are easy to compute (Ruge-Murcia 2020); and
vi) LPIRFs allow us to distinguish the responses of trading volume to return shocks in different return regimes. Thus, the results may provide richer insights into the case of investor behavior and may allow more realistic policy suggestions considering the effect of the existing regime.

### Unit root tests

First, we calculated the return series using Eq. (1):

1 $$R = lnBIST100_{t} - lnBIST100_{t - 1}$$

Here R denotes the returns, (*ln*BIST100)_t_ represents the closing price of the BIST100 index at time *t*, and (*ln*BIST100)_(t-1)_ represents the closing price at time *t−*1. To test the stability of the variables, we applied the augmented Dickey-Fuller (ADF) (Dickey and Fuller 1979) test, which is shown in Eqs. (2) and (3):

2 $$\Delta y_{t} = \alpha + \beta T + \varphi y_{t - 1} + \sum\limits_{i = 1}^{k} {\delta_{i} \Delta y_{t - 1} + u_{t} }$$

3 $$\Delta y_{t} = \alpha + \varphi y_{t - 1} + \sum\limits_{i = 1}^{k} {\delta_{i} \Delta y_{t - 1} + u_{t} }$$

In Eq. (2), the deterministic term, T, refers to the trend. The ADF is based on testing the hypothesis, H_0_: δ = 0, against the alternative hypothesis, H_a_: δ < 0 (Alp and Seven 2019). Because the null hypothesis in the ADF test is for the presence of a unit root, we apply another test, the KPSS (Kwiatkowski et al. 1992), which assumes that the variables are stationary and provides more consistent results. The KPSS test statistics depend on the error terms obtained from the regressions of the time series with external variables. We also prefer the KPSS test because it is equally effective in detecting the presence of unit roots for both linear and nonlinear time series. Considering the applied unit-root test results, we included the return series^5^ at its level and the trading volume by taking the logarithmic difference.

### Granger causality test and FFNN, nonlinear granger causality test

Granger (1969) first suggested the causality test. Accordingly, for two time series that are stationary, e.g., X and Y, if the results of forecasting future information about the Y series depend more on the historical information of the X series rather than that of the Y series, the X series is the Granger cause of the Y series. To apply the Granger causality test, a VAR model is first established (Ren et al. 2020):

4 $$Y_{t} = \sum\limits_{i = 1}^{p} {\alpha_{i} Y_{t - i} + \varepsilon_{Y,t} }$$

5 $$Y_{t} = \sum\limits_{i = 1}^{p} {a_{i} X_{t - i} + b_{i} Y_{t - i} + \varepsilon_{{Y\left| {X,t} \right.}} }$$

In Eqs. (4) and (5), p refers to the dimension of the VAR model, α_i_, a_i_, and b_i_ refer to the coefficients of the model, and ${\varvec{\varepsilon}}_{{{\varvec{Y}},{\varvec{t}}}}$ and ${\varvec{\varepsilon}}_{{{\varvec{Y}}\left| {{\varvec{X}},{\varvec{t}}} \right.}}$ denote the residual error terms. Whether there is Granger causality from X to Y can be seen by comparing the residual errors in Eqs. (4) and (5). The Granger causality index (GCI) can be calculated using the following equation (Ren et al. 2020):

6 $$GCI_{X \to Y} = \ln \frac{{{\text{var}} \left( {\varepsilon_{Y,t} } \right)}}{{{\text{var}} \left( {\varepsilon_{{Y\left| {X,t} \right.}} } \right)}}$$

If, ${\varvec{\varepsilon}}_{{{\varvec{Y}}\left| {\varvec{X}} \right.}}$ < ${\varvec{\varepsilon}}_{{\varvec{Y}}}$, then ${\varvec{GCI}}_{{{\varvec{X}} \to {\varvec{Y}}}}$ > 0, and this indicates that X is the Granger cause of Y. The classic Granger causality test described above is based on linear models.

**Fig. 1:**
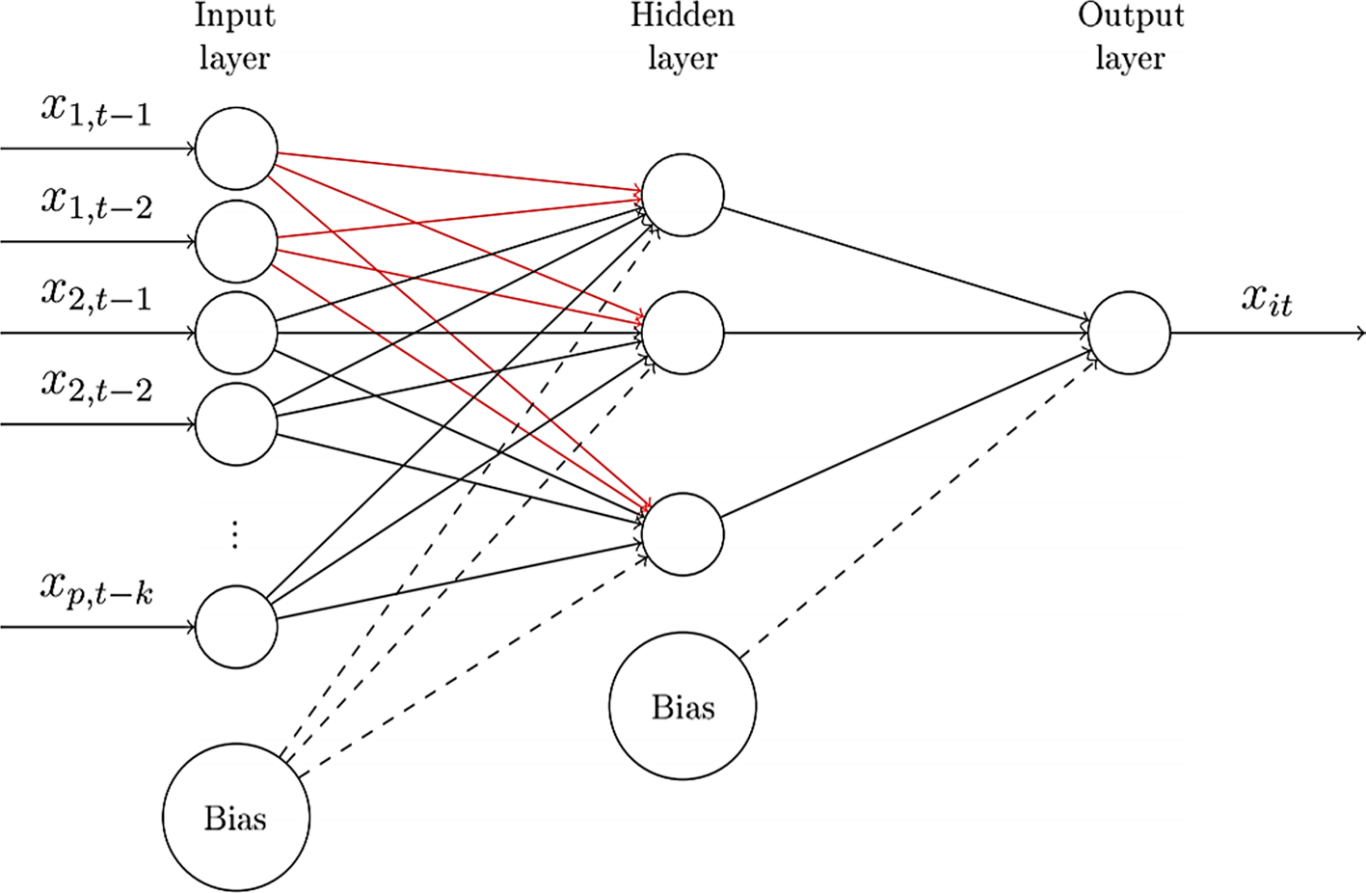
Feed-forward neural network nonlinear Granger causality. *Reference*: Calvo-Pardo et al. (2021)

An NN comprises neurons lined up between layers (Montalto et al. 2015). The first is the "input layer" that receives external inputs, and the last layer is the "output layer," which provides the results of the measurement for the entire network. All layers between the input and output layers are called "hidden layers.^6^" Fig. 1 illustrates a virtual demonstration of the process behind the Granger causality test based on a multi-variable FFNN with one hidden layer (Calvo-Pardo et al. 2021).

Several different methodologies to capture causal relationships between variables have been proposed and applied for various purposes in the scientific literature. According to Li et al. (2022), financial datasets follow complex distributions and therefore linear techniques may not be useful in many cases. Kou et al. (2014) argued that no algorithm could achieve the best performance on all measurements for any dataset, and more than one single performance measure must be utilized to evaluate clustering algorithms. However, in Li et al.’s (2022) study, for the purpose of clustering, the Ada-Ellip algorithm is suggested because it outperforms several other algorithms. Meanwhile, Kou et al. (2021a) proposed a hybrid method as a multi-criterion, decision-making (MCDM) technique that covers the application of DEMATEL, TOPSIS, and VIKOR to evaluate Fintech-based investments in European banking services. In addition to weighting the criteria, the cause-and-effect relationships between these items are also investigated through the abovementioned models. In another study (Kou et al. 2022), an integrated methodology is suggested that includes incomplete preferences, group decision-making with consensus, spherical fuzzy sets, and DEMATEL to evaluate the complex decision-making approach for solar, energy-based, transportation investment projects. To evaluate the predictive power of payment and transactional data in bankruptcy prediction, Kou et al. (2021b) employed commonly used classifiers (i.e., linear discriminant analysis (LDA), logical regression (LR), support vector machine (SVM), decision tree (DT), random forest (RF), XGBoost (XGB), and NN) in five different models. In the case of MCDM using either financial data or non-financial data, the nature of complex systems and distributions of data require nonlinear and complex methods to understand real-life issues. In the overconfidence-bias literature, the relationship between return and trading volume has been commonly investigated through various methods. Because observations of two variables do not require an MCDM process, in our study, FFNNs are used for clustering and weighting to capture causality in the complex nature of financial data.

### Nonlinear IRFs

Jordà (2005) suggested estimating ordinary least squares (OLS) regressions for each forecast horizon as the first step:

7 $$y_{t + h} = \alpha^{h} + B_{1}^{h} y_{t - 1} + \cdots + B_{p}^{h} y_{t - p} + u_{{t + h^{\prime}}}^{h} h = 0,1, \ldots ,H - 1,$$

where ${\varvec{\alpha}}^{{\varvec{h}}}$ is the vector of constants and ${\varvec{B}}_{1}^{{\varvec{h}}}$ are parameter matrices for lag p and forecast horizon h. The vector elements, $u_{t + h}^{h}$, are autocorrelated and/or heteroscedastic disturbances. The collection of all regressions of Eq. (7) are called LPs. The slope matrix, ${\varvec{B}}_{1}^{{\varvec{h}}}$, can be interpreted as the response of ${\varvec{y}}_{{{\varvec{t}} + {\varvec{h}}}}$ to a reduced-form shock in t (Kilian and Kim 2011). Structural impulse responses are estimated using the following equation:

8 $$\widehat{IR}\left( {t,h,d_{i} } \right) = \hat{B}_{1}^{h} d_{i} ,$$

where ${\varvec{d}}_{{\varvec{i}}} = {\varvec{B}}_{0}^{ - 1}$ and the shock matrix, ${\varvec{d}}_{{\varvec{i}}}$, must be identified from a linear VAR. To extend the LPs to nonlinear frameworks, the data are split into two regimes using a dummy variable. Auerbach and Gorodnichenko (2012) propose computing state probabilities using a logistic function that allows using all observations for the estimations (Adämmer 2019). The logistic function yields the following:

9 $$F\left( {z_{t} } \right) = \frac{{e^{{\left( { - \gamma z_{t} } \right)}} }}{{\left( {1 + e^{{\left( { - \gamma z_{t} } \right)}} } \right)^{^{\prime}} }}$$

10 $${\text{var}} \left( {z_{t} } \right) = 1, E\left( {z_{t} } \right) = 0,$$

where ${\varvec{z}}_{{\varvec{t}}}$ is standardized so that γ (> 0) is scale-invariant. Auerbach and Gorodnichenko (2013) suggest standardizing the cyclical components of the filter based on the method that Hodrick and Prescott (1997) introduced to obtain the variable, ${\varvec{z}}_{{\varvec{t}}}$. As Adämmer (2019) stated, the observations for the two regimes are the products of the transition function and endogenous variables:

11 $$Regime{ }\;1 \left( {R_{1} } \right):y_{t - l} .\left( {1 - F\left( {z_{t - 1} } \right)} \right), l = 1, \ldots ,p,$$

12 $$Regime\;2 \left( {R_{2} } \right):y_{t - l} .F\left( {z_{t - 1} } \right), l = 1, \ldots ,p,$$

Structural, nonlinear impulse responses can be estimated using the following equations:

13 $$\widehat{IR}^{{R_{1} }} \left( {t,h,d_{i} } \right) = \hat{B}_{{1,R_{1} }}^{h} d_{i} h = 0, \ldots ,H - 1,$$

14 $$\widehat{IR}^{{R_{2} }} \left( {t,h,d_{i} } \right) = \hat{B}_{{1,R_{2} }}^{h} d_{i} h = 0, \ldots ,H - 1,$$

where $\hat{B}_{1,R1}^{0} = I$ and $\hat{B}_{1,R2}^{0} = I$. The coefficients matrices, $\hat{B}_{1,R1}^{h}$ and $\hat{B}_{1,R2}^{h}$, are obtained from the following LPs:

15 $$\begin{aligned} y_{t + h} = & \;\alpha^{h} + B_{{1,R_{1} }}^{h} \left( {y_{t - 1} \cdot \left( {1 - F\left( {z_{t - 1} } \right)} \right)} \right) + \ldots + B_{{p,R_{1} }}^{h} \left( {y_{t - p} \cdot \left( {1 - F\left( {z_{t - 1} } \right)} \right)} \right) \\ & + B_{{1,R_{2} }}^{h} \left( {y_{t - 1} \cdot F\left( {z_{t - 1} } \right)} \right) + \ldots + B_{{p,R_{2} }}^{h} \left( {y_{t - 1} \cdot F\left( {z_{t - 1} } \right) + u_{{t + h^{\prime}}}^{h} } \right) \\ \end{aligned}$$

$$h = 0, \ldots ,H - 1$$

Many studies have applied LPIRFs for different cases in the literature (see Ahmed and Cassou 2016; Kuiper and Lansink 2013; Montes-Rojas 2019).

## Empirical results and discussion

### Findings for overall period

#### Preliminary tests

Table 1 presents the variables’ descriptive statistics. The average daily trading volume was 1377,835, while the average daily return was 0,0001. The trading volume reached a maximum value of 3249,239, while the maximum value of the return was 0,0581. The minimum values were 220,7868 and − 0,0841 for the trading volume and return, respectively. Skewness and kurtosis values should be examined to determine whether the variables fit the normal distribution. In the normal distribution, the value of kurtosis should be equal to 3, while the skewness value should be 0. From Table 1, the kurtosis values for both variables are greater than 3. Therefore, we conclude that the series exhibit fat tail distributions. Moreover, the trading volume is skewed to the right, with a skewness value greater than 0, and the return, with a skewness value smaller than 0, is skewed to the left. In other words, both series do not follow a normal distribution.

**Table 1:** Descriptive statistics

	Daily trading volume (TO) (Million USD)	Daily return (R)
Mean	1377	0.0001
Median	1350	0.0006
Maximum	3249	0.0581
Minimum	220	− 0.0841
Std. Dev	407.51	0.0135
Skewness	0.5112	− 0.6448
Kurtosis	3.7216	6.6438
Jarque–Bera	88.0513	818.0164
Probability	0	0
Total	1858.700	0.227519
Number of obs	1349	1314

**Fig. 2:**
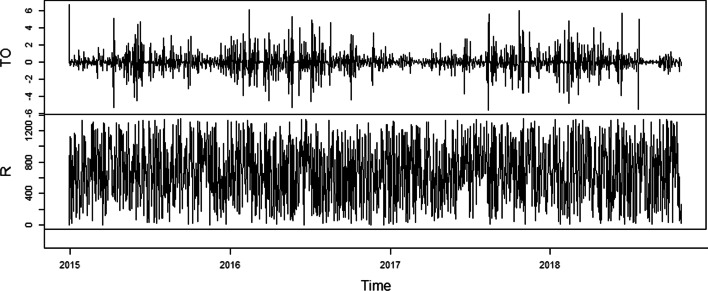
Trading volume and return series of BIST100 (01.01.2015–05.12.2020)

In Fig. 2, the upper panel shows the trading volume while the lower panel shows the return series. Clearly, there are ups and downs in certain periods in both series, particularly in the trading volume. Table 2 presents the results of the ADF and KPSS unit-root tests. The return variable was stationary according to both unit root tests; however, the trading volume contains a unit root at the level and becomes stationary at its first difference.

**Table 2:** ADF and KPSS unit root test results

ADF			KPSS		
v	*P*-value	Test statistics		*P*-value	Test statistics
Ln(TO)	0.001	− 3.759	Ln(TO)	0.01	2.527
Ln(TO)-1. first difference	0.001	− 49.1529	Ln(TO)-1. First Difference	0.1	0.0016
R	0.001	− 36.9326	R	0.1	0.0548

#### AI application of granger causality and IRFs

Table 3 presents the results of the standard Granger and FFNN, nonlinear Granger causality tests. We determined lag length based on the Akaike information criterion^7^ (AIC). The probability values of the F-test are smaller than 0.05, which implies that there is a causality from the return to the trading volume. Accordingly, the null hypothesis that the return is not the Granger cause of the trading volume is rejected at the 5% significance level. The direction of causality is only from the return to the trading volume. The findings indicate that the BIST100 investors exhibit overconfidence behavior.^8^ Thus, for the first hypothesis, we fail to reject the null hypothesis.

**Table 3:** The results of granger causality tests

*Granger causality test*	
Lag parameter	*P* = 8
Granger causality index	GCI = 0.0211
F-test value	3.6602
F-test *P*-value	0.0003
5% critical value of risk	2.032
*Nonlinear Granger causality test results*	
Lag parameter	*P* = 8
Granger causality index	GCI = 0.0782
F-test value	107.226
F-test *P*-value	3.27606e-24
5% critical value of risk	3.936

##### Linear impulse-response analysis

**Fig. 3:**
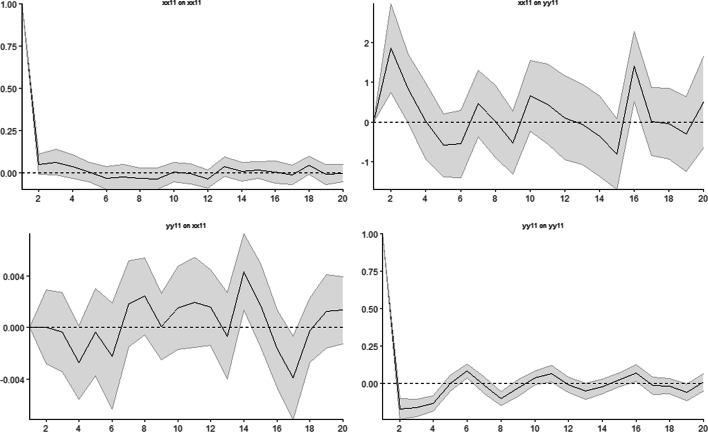
Linear impulse response analysis. *Note*: Grey shaded area illustrates 95% confidence intervals

Having detected the causality between return and trading volume, we applied linear and nonlinear impulse-response analyses based on LP to examine the response of the trading volume to shocks occurring to the return. According to the results of the linear IRFs in Fig. 3, in the upper right corner of the panel, a one-unit shock to the return has an impact on the trading volume. On the 1st and 2nd days, the trading volume increases by approximately 2% when a one-unit shock occurs to the return. Between the 2nd and the 5th days, the magnitude of the response of the trading volume to the shocks to the return declines, while on the 6th and 7th days, the response increases again. Forecast horizons for impulse responses for 20 days show that the trading volume responds positively and negatively to shocks to the return, varying daily, and the response persists.

##### Nonlinear impulse-response analysis

**Fig. 4:**
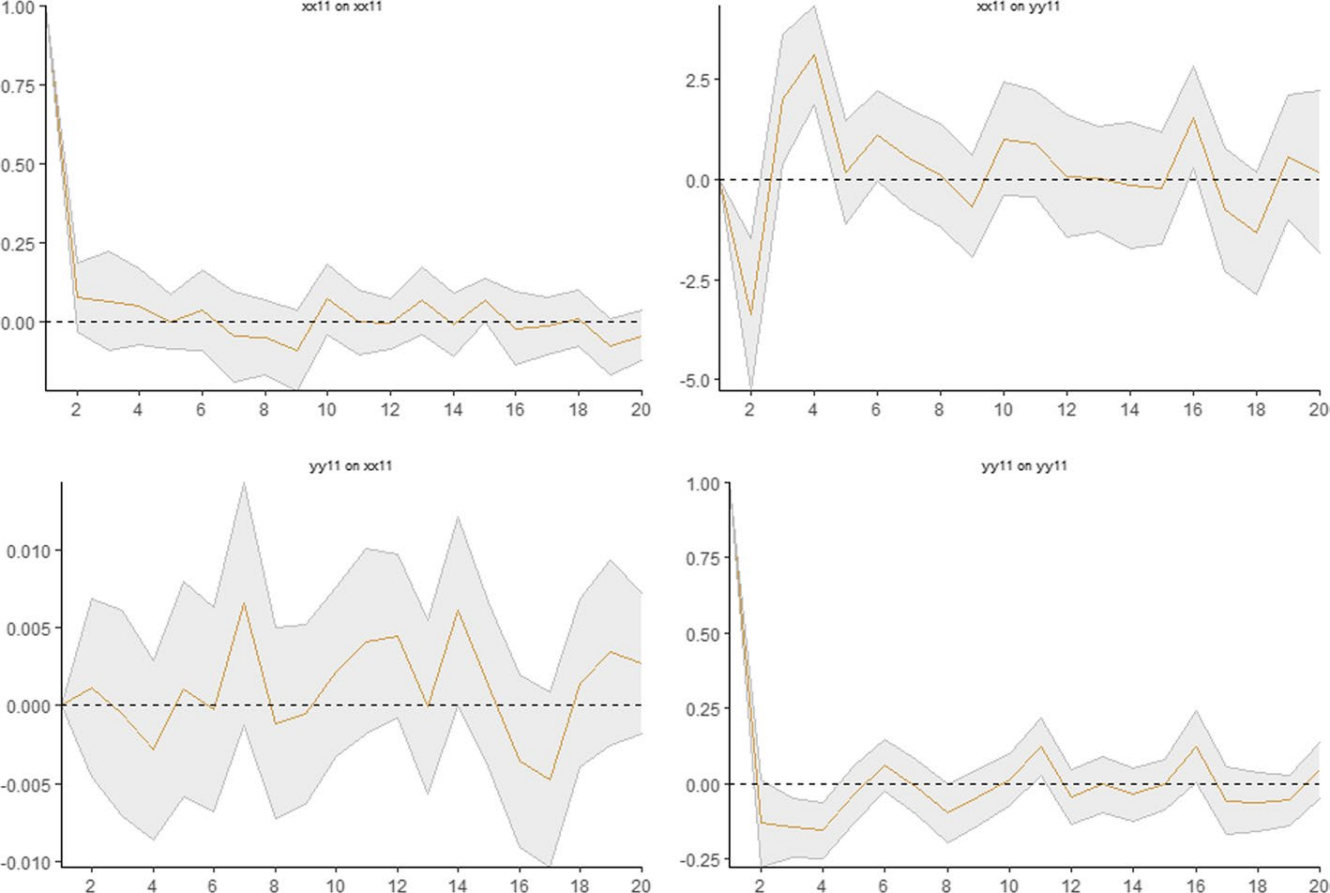
Impulse response analysis in the low-return regime. *Note*: Grey shaded area illustrates 95% confidence intervals

**Fig. 5:**
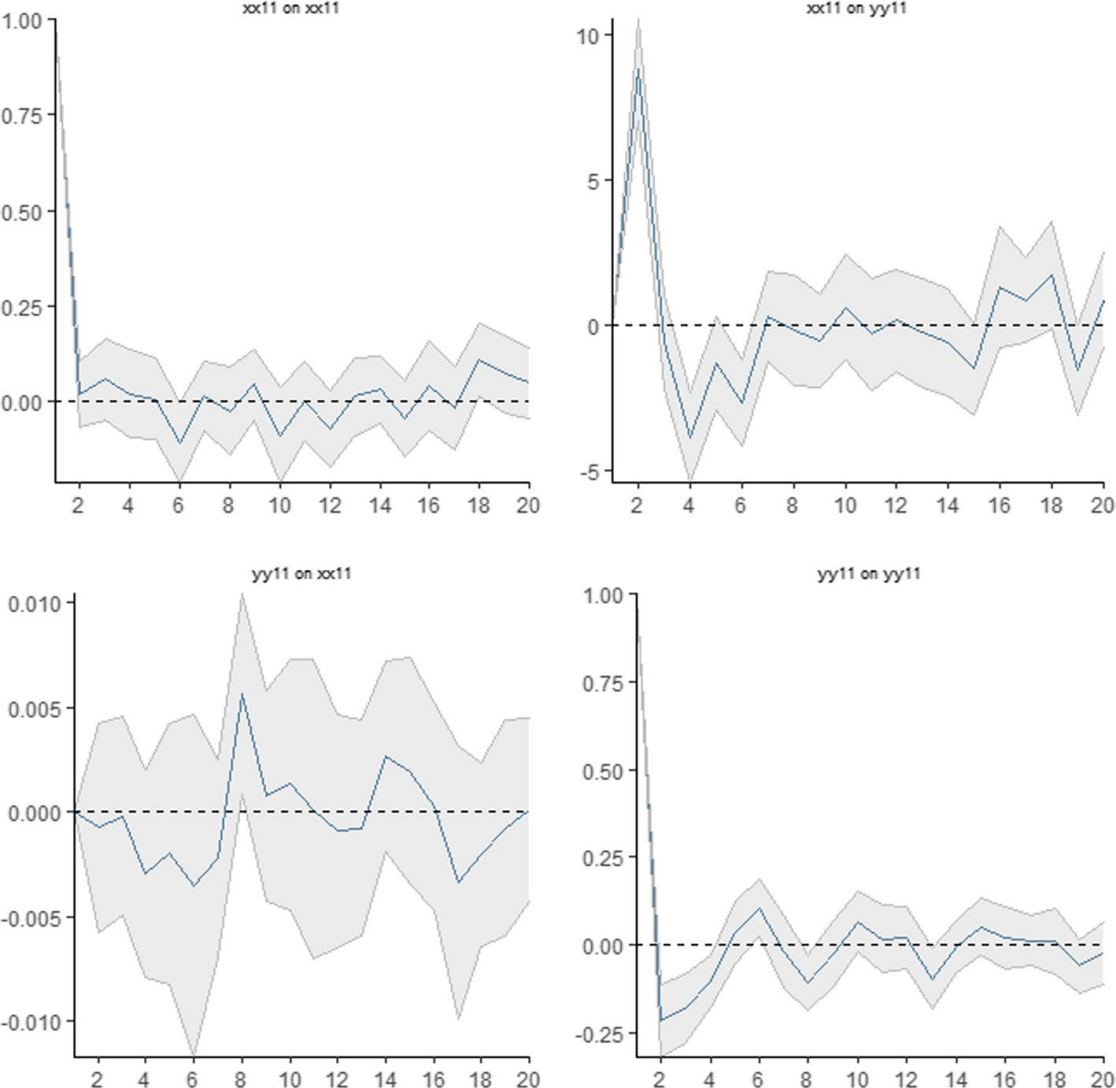
Impulse response analysis in the high-return regime. *Note*: Grey shaded area illustrates 95% confidence intervals

In the LPIRFs, two different market regimes with low and high returns were determined. Figures 4 and 5 show the results of the LPIRFs.^9^ In both regimes, the trading volume quickly reacts to the shocks that occur to the return; the first major reaction of the trading volume to the return shocks usually lasts for 2 days; the reaction is stronger in the first 2–4 days, while in the following days, the response remains lower than that in the first days. It is also seen that the response of the trading volume to the return shocks lasts for 20 days^10^ and persists in both the low- and high-return regimes. This finding contradicts studies that employed linear IRF analysis. Therefore, for the second hypothesis, we reject the null hypothesis.

##### Low-return regime

Figure 4 shows the change in the response of the trading volume when a one-unit shock occurs to the return in the low-return regime. In Fig. 4, the upper right corner of the panel shows that a one-unit shock to the return in the low-return regime leads to a decrease in the trading volume on the 1st day (3%), and overconfidence bias occurs on the 2nd day as the response of the trading volume to the return shock increases on the 4th day (3%). The response of the trading volume to the return shock is mostly positive, implying that the trading volume responds to the return shock in the same direction. This finding indicates that overconfidence behavior is more persistent in the low-return regime. We may speculate that investors do not find the amount of return sufficient and seek higher returns in the low-return regime. This may increase their risk appetite and may therefore result in excessive trading because overconfident investors do not hold stocks for long, often monitor their profits, and trade more frequently (Abreu and Mendes 2012; Strahilevitz et al. 2015; Tourani-Rad and Kirkby 2005). Another possible explanation for this finding may be the lower uncertainty associated with the lower volatility in the low-return regime, which makes overconfident investors think that the prices in this regime are more predictable.

##### High-return regime

Figure 5 shows the change in the response of the trading volume when a one-unit shock occurs to the return in the high-return regime. The upper right corner of Fig. 5 shows that a one-unit shock to the return in the high-return regime increases the trading volume by 9% in total on the 1st and 2nd days; the response of the trading volume decreases and overconfidence disappears until the 16th day. After the 3rd day, the response of the trading volume to the return shock becomes and mostly stays negative, implying that the trading volume mostly changes in the opposite direction to that of the shock to the return and decreases (increases) after a positive (negative) return shock. We may speculate that investors tend to keep their returns and avoid more trading after positive returns from the high-return regime, which also implies that overconfidence behavior is strong only on the first day and then becomes more moderate in this regime. Meanwhile, after a negative return shock (decrease in return), the trading volume increases, and this reaction may be the motivation for compensation for previous losses.^11^ Moreover, when we compare the two graphs for the low- and high-return regimes, the responses of the trading volume to the return shocks exhibit asymmetries in the different regimes; therefore, we fail to reject the third null hypothesis of the study. In the low-return (high-return) regime, the highest response of the trading volume to a one-unit shock is approximately 2.75% (9%). The magnitude of the response in the low-return regime is lower than that in the high-return regime. However, during the 20-day forecasting horizon, the responses of the trading volume to the shocks to the return are mostly positive in the low-return regime, in contrast to those in the high-return regime. Therefore, we conclude that overconfidence is more persistent and stronger in the low-return regime after the 2nd day. Based on the overall empirical models, we fail to reject the nulls for Hypotheses 1 and 3 and reject Hypotheses 2 and 4.

As mentioned above, the magnitude of the first response (1st day) of the trading volume to the return shock is greater in the high- than in the low-return regime. However, after the first-day, overconfidence weakens in the high-return regime and becomes moderate rather than extreme, and is more persistent in the low-return regime. In other words, if the past return is positive but low, overtrading is stronger; however, if the past return is positive and high, overtrading behavior is not strong. This finding suggests that overconfident investors in the BIST100 are mostly motivated by lower returns, while higher returns cause the investors to be moderate.^12^ Liu et al. (2016) employed a double-threshold generalized autoregressive conditional heteroscedasticity (GARCH) model and found evidence of more trading activity by overconfident investors in high market-return regimes than in low ones. According to the authors, the intuition underlying this evidence is that high market gains induce investors to trade overconfidently to a larger extent than low market gains. However, by employing a different methodology, we obtained contradictory results, as some studies claim that overconfidence is likely to be strong and detectable in a bull market (e.g., Chuang and Lee 2006; Daniel et al. 1998, 2001; Gervais and Odean 2001; Gao et al. 2021; Huang et al. 2022) because overconfidence is stimulated by realized past success and is associated with high returns, according to Jlassi et al. (2014). However, our results indicate that investors may be intimidated by experiencing losses after more trading in the high-return regime, which is more volatile (Liu et al. 2016). Investors may presume that high returns based on price spikes in volatile periods may also result in high losses based on sudden price declines, which supports a more moderate type of overconfidence. Therefore, a high-return regime does not induce overconfidence behavior in BIST. According to our results, an increase in return—investors’ past success—after more trading occurs only in the low-return regime and therefore investors are motivated to be overconfident in the low-return regime. Contrary to the results of the abovementioned studies, a low return induces overconfidence behavior in BIST. According to Bajzik (2021), the impact of trading volume on stock returns in emerging stock markets has the opposite sign to that on returns in developed markets. This may be a possible reason for the contradictory results of our analysis compared to those of the abovementioned studies. Another reason may be the rich outcomes of the selected empirical methodology in our study, which is the first application in the overconfidence-bias literature. Based on the findings for the first day, overconfidence appears to be stronger in the high-return regime; however, the forecast horizon shows the subsequent changes in the responses of the trading volume, which demonstrates the reverse. Similarly, Liu et al.’s (2016) static models may produce results that contradict those of a dynamic model. Thus, employing LPIRFs in our study contributes to the existing knowledge on overconfidence bias in stock markets.

### Findings for subperiods

**Fig. 6:**
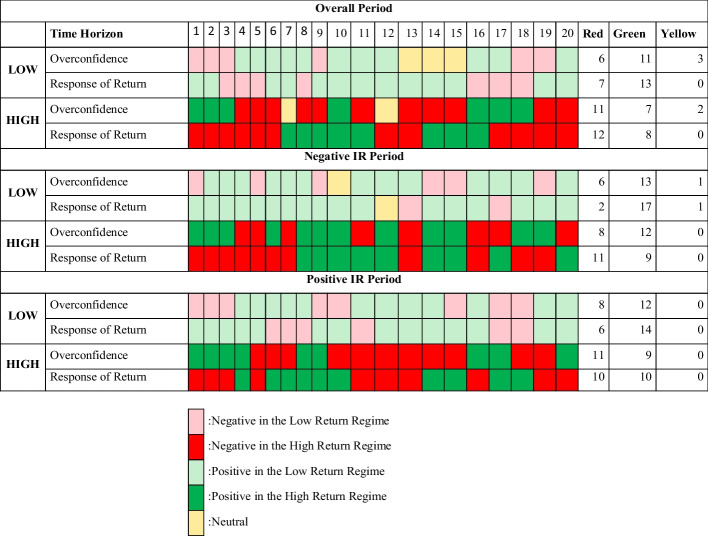
The change in overconfidence and in the response of returns. *Note*: Positive means the existence of overconfidence while negative means that overconfidence doesn’t exist. For “response of return”, positive means that the line is above zero or negative if the line is below zero (x-axis)

**Fig. 7:**
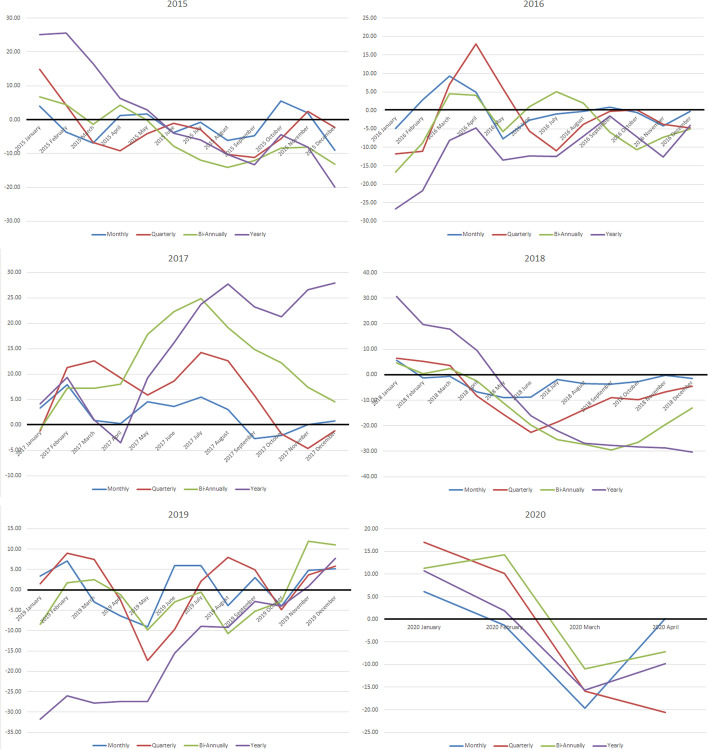
The real rate of return in BIST100 during January/2015-April/2020. *Source***:** Turkish Statistical Institute **(**TurkStat), 2021. (https://turkstatweb.tuik.gov.tr/PreTablo.do?alt_id=1064, accessed on 11.05.2021)

**Fig. 8:**
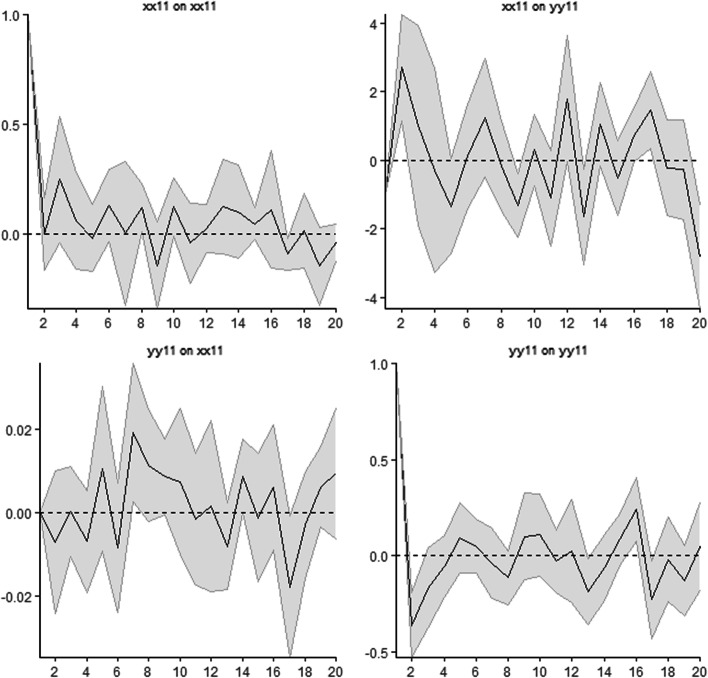
The subperiod analysis of linear IRF for the period in which CPI > IR (Negative interest rates) (12.02.2019–05.12.2020)

**Fig. 9:**
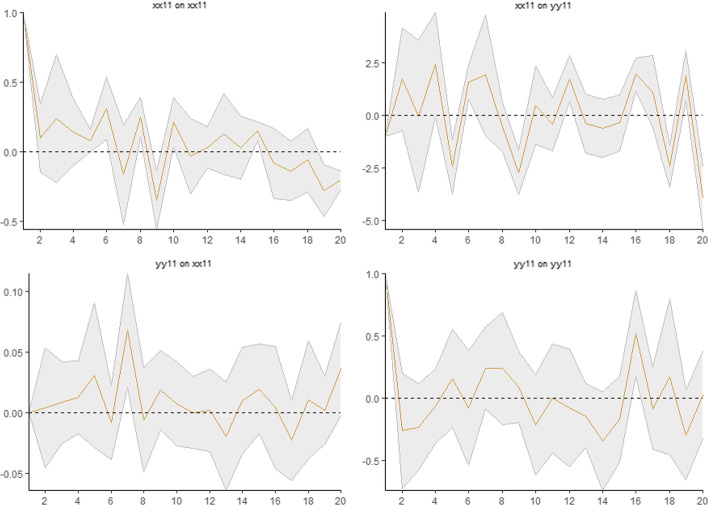
The subperiod analysis of low-return regime for the period in which CPI > IR (Negative interest rates) (12.02.2019–05.12.2020)

**Fig. 10:**
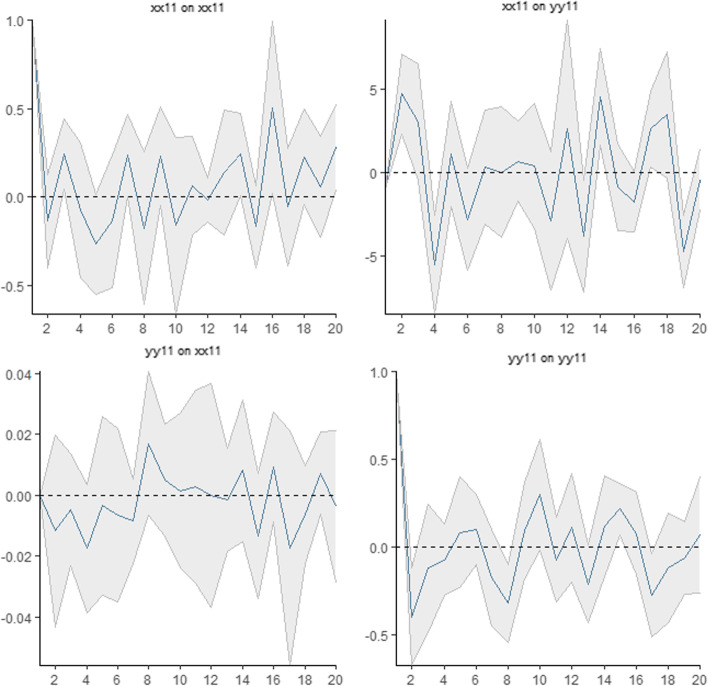
The subperiod analysis of high-return regime for the period in which CPI > IR (Negative interest rates) (12.02.2019–05.12.2020). *Note*: Grey shaded area illustrates 95% confidence intervals. The figures from top to bottom correspond to linear analysis, low return regime, high return regime respectively

**Fig. 11:**
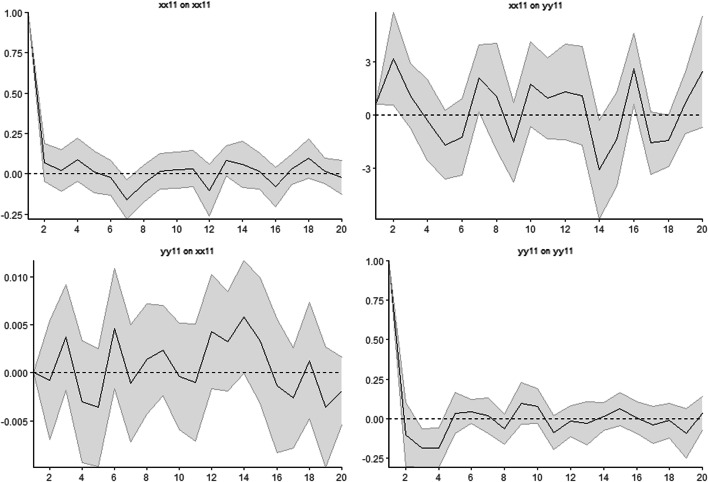
The subperiod analysis of linear IRF for the period in which CPI < IR (Positive interest rates) (11.01.2018–11.29.2019)

**Fig. 12:**
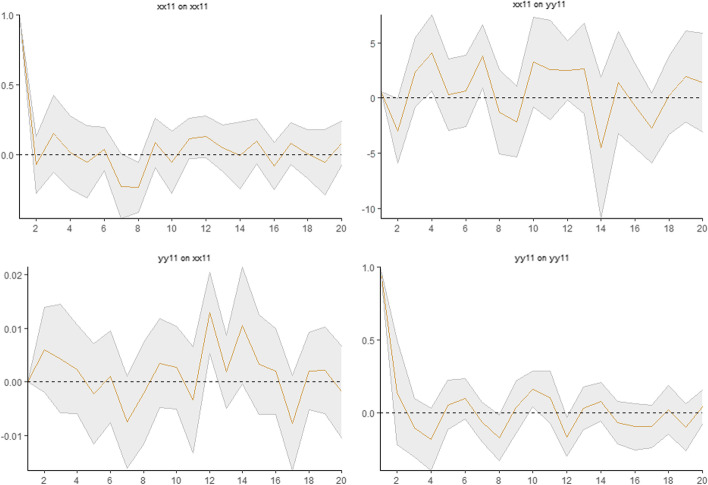
The subperiod analysis of low-return regime for the period in which CPI < IR (Positive interest rates) (11.01.2018–11.29.2019)

**Fig. 13:**
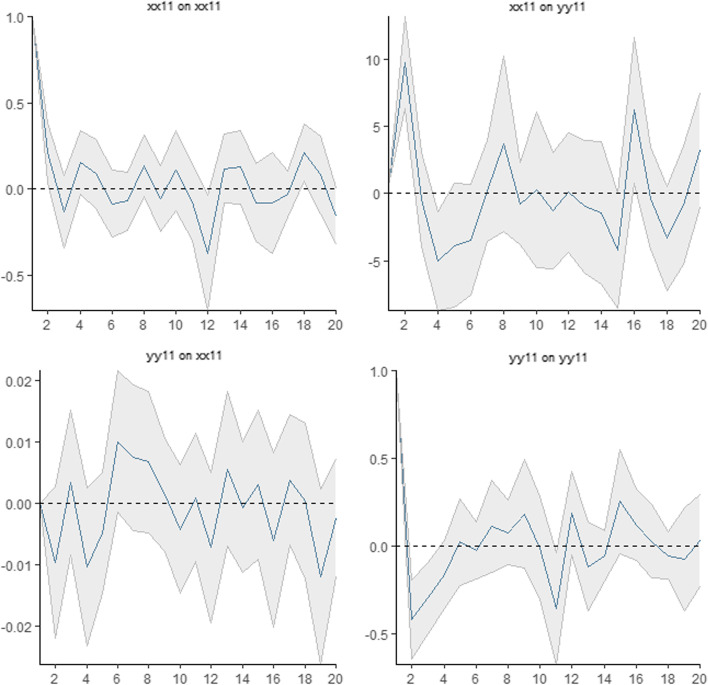
The subperiod analysis of high-return regime for the period in which CPI < IR (Positive interest rates) (11.01.2018–11.29.2019). *Note*: Grey shaded area illustrates 95% confidence intervals. The figures from top to bottom correspond to linear analysis, low return regime, high return regime respectively

We also divided our period into two subperiods considering negative/positive interest rates to investigate whether investors’ overconfidence changes in the low-/high-return regimes when they need to avoid losses resulting from negative interest rates (the subperiod results can be found in Appendix C, Figs. 8, 9, 10, 11, 12, 13). Our subperiod analysis supported the overall findings of the study; however, when interest rates were negative during the first subperiod (12.02.2019–05.12.2020), investor overconfidence was greater, stronger, and more persistent in the high-return regime. We may speculate that in the high-return regime, investors’ overconfidence becomes stronger only when they face losses because of the negative interest rates and their increased willingness to take more risks by overtrading. Another interesting finding is that in the low-return regime, investors’ overconfidence is greater for the positive interest-rate subperiod (11.01.2018–11.29.2019) than for the overall period (by approximately 4%). This finding also implies that when the investment environment is more stable, the volatility and uncertainty levels are low, overconfident investors become strongly overconfident, and they may think that prices are more predictable in the low-return regime (Fig. 6 illustrates the overall findings).

#### Responses of return to trading volume shocks

In addition to the hypotheses that have been tested in the study, another interesting finding is that the trading volume influences the return through 20 days (see both the linear and nonlinear test results on the bottom left side of the impulse-response graphs). In the low-return regime, the response of the return to shocks to the trading volume is mostly positive, while it is mostly negative in the high-return regime. Thus, an increase in the trading volume results in an increase in the return in the low-return regime; however, contrarily, the return decreases after an increase in the trading volume in the high-return regime. This finding also confirms the previous findings that implied that overconfidence was more persistent in the low-return regime. Accordingly, an increase in return after an increase in trading volume in the low-return regime induces investors’ overconfidence based on the realized past success. Moreover, this finding may be a possible explanation for investors’ opposite behaviors based on the responses of returns to trading volume shocks in different regimes. More trading results in a decrease in return in the high-return regime; therefore, investors do not increase trading volume after they earn a positive return and probably keep holding the same portfolios, which also implies that overconfidence behavior becomes more moderate in the high-return regime. The subperiod analysis also supported the findings of the overall analysis. Furthermore, the return positively responded to the shocks to the trading volume almost daily in the low-return regime during the negative interest-rate subperiod. Despite the results of the Granger causality test, the results of the LPIRFs suggest that trading volume can predict future stock market returns in BIST, contrary to the efficient market hypothesis.

#### Summary of the findings

In a nutshell, by applying the abovementioned models, we obtained interesting results:

1. Overconfidence bias exists in BIST during the observation period of this study (2015–2020 and subperiods). Our results are consistent with those of studies on emerging markets, including BIST.
2. Overconfidence behavior shows persistence for at least 20 days. This result contradicts those of studies that employed linear IRFs (e.g., Alsabban and Alarfaj 2020; Metwally and Darwish 2015; Sheikh and Riaz 2012; Statman et al. 2006; Zaiane and Abaoub 2009).
3. The duration and degree of overconfidence behavior vary based on the return regime, confirming the asymmetries between regimes.
4. The first response of the trading volume in the low-return regime is negative in all cases, implying that overconfidence occurs with a delay in this regime.
5. In the low-return regime, the strongest overconfidence is observed in the positive interest-rate subperiod (4%). The degree of overconfidence is lower in the overall period (2.75%) and in the negative interest-rate subperiod (2.4%). Meanwhile, in the high-return regime, the strongest overconfidence is observed in the overall period and in the positive interest-rate subperiod (9%). However, it is smaller (4%) but persistent in the negative interest-rate subperiod. In sum, in the negative interest-rate period, a high-return regime induces overconfidence behavior, whereas in the positive interest-rate period, a low-return regime induces overconfidence behavior.
6. For the overall period, the overconfidence behavior is stronger in the high-return regime on the first three days; however, it weakens thereafter; in the low-return regime, the degree of overconfidence is lower, although it persists for 20 days.
7. Overconfidence behavior becomes more persistent only in the negative interest-rate subperiod; in the high-return regime, however, the response of the return to the trading volume shock is mostly negative.
8. In both the overall period and the subperiods, in the low-return regime, the response of the return to the trading volume shock is mostly positive, implying that in the relevant regime, overconfident investors’ overtrading results in positive returns. In the high-return regime, overtrading does not result in positive returns, regardless of the negative/positive interest rates. As discussed in the literature review section, overconfidence may be advantageous or disadvantageous, depending on market conditions. Thus, when interest rates are negative in a low-return regime in the stock market, overconfidence results in positive returns (advantageous). However, when interest rates are positive and the market is in a high-return regime, overconfidence results in negative returns (disadvantageous). This finding implies that overconfidence may result from some rational motivations on its merits.

Figure 6 comparatively illustrates the dominance of overconfidence and positive/negative returns in the low- and high-return regimes in all the three periods. The most permanent overconfidence occurs when interest rates are negative in the low-return regime. This outcome implies that overconfidence may result from some rational purposes such as avoiding losses in relevant market conditions.

## Concluding remarks

As it is widely accepted in the literature, economic activities such as investment decisions are driven by many psychological factors, rather than by a rational process supported by perfect knowledge, which is impossible. Overconfidence is a psychologically driven force that results in inefficiencies and crises in financial markets, which must be investigated. In this study, we examined whether investors exhibited overconfidence behavior in the BIST100 in the period 01.01.2015–05.12.2020. As a key contribution to the literature gap, we employed a nonlinear Granger causality test based on an FFNN and nonlinear LPIRFs to detect the causality from the return to the trading volume and the responses of the latter to return shocks in different return regimes. To the best of our knowledge, this is the first application of the above models to examine overconfidence bias. Additionally, we analyzed two subperiods based on negative/positive interest rates to better understand the motivations behind overconfidence behavior in BIST.

Based on the asymmetric responses of trading volume to return shocks in different return regimes, investors and portfolio managers should consider the return regime in the market to estimate the dominant behavior. During a low-return regime, investors who seek higher returns dominate the market, which results in overconfidence behavior and market inefficiencies. Moreover, a positive response of the return to shocks to the trading volume will not result in portfolio gains for all investors in a low-return regime. Thus, overconfident investors with risky preferences may face further losses.^13^ The results of the LPIRFs implied that the responses of return to shocks to trading volume changed daily. Therefore, investors should be more patient rather than aggressive, and should consider portfolio diversification and hedging. Investors should be aware that overconfident investors exist in the market, and that during a low-return regime, this type of investor dominates and influences the returns through aggressive and excessive trading.

As a policy suggestion, investors may be trained in the fundamentals of investing in a stock market. Moreover, developing a warning system consistent with the trading sentiment could help investors behave more prudently during a low-return regime. It is proposed that policies that will contribute to the development of financial literacy will also help investors make more rational decisions rather than judgmental decisions such as overconfidence.

For future research, the dataset may be extended to include other emerging and advanced stock markets for comparison. Additionally, subperiods such as the Covid-19 period may be separately analyzed (see Apergis 2022; Kuranchie-Pong and Forson 2022). Another contribution may be the use of high-frequency data such as hourly data to observe investors’ more rapid reactions after market gains. Applying the same methodologies to different stock markets may also shed light on the dynamic reactions of overconfidence in different return regimes.

## Data Availability

The datasets generated and/or analysed during the current study are available in the [Website of GitHub] repository, [https://github.com/behaviouralfinancier/overconfidenceAI].

## Appendix A

(See Table 4).

**Table 4:** BDS test results of return and trading volume

Return (R)				
	[0.0067]	[0.0135]	[0.0202]	[0.027]
*Standard normal*				
2	3.3432	3.8973	4.0242	4.007
3	5.2167	5.4117	5.252	5.112
*P value*				
2	8.00E−04	1.00E−04	1.00E−04	1.00E−04
3	0.00E+00	0.00E+00	0.00E+00	0.00E+00

Trading volume (TO)				
	[0.1146]	[0.2292]	[0.3438]	[0.4584]
*Standard normal*				
2	6.0773	6.4588	7.2268	7.6168
3	7.2815	7.6552	8.1736	8.4619
*P value*				
2	0	0	0	0
3	0	0	0	0

## Appendix B

(See Fig. 7).

## Appendix C

(See Figs. 8, 9, 10, 11, 12, 13).

## Abbreviations

AI: Artificial intelligence
AIC: Akaike information criteria
ANN: Artificial neural networks
BIST: Borsa İstanbul
CEO: Chief executive officer
CRA: Central registry agency
DT: Decision tree
FFNN: Feed-forward neural network
GCI: Granger causality index
IRF: Impulse response function
LDA: Linear discriminant analysis
LP: Local projections
LPIRF: Local projections impulse response functions
LR: Logical regression
MCDM: Multi-criteria decision-making
RF: Random forest
SVAR: Structural vector autoregression
SVM: Support vector machine
US: United States
VAR: Vector autoregression
XGB: XGBoost

## Footnotes

1 For studies that examined the impact of bear/bull market conditions on overconfidence behaviour, see (Gupta et al. 2018; Jlassi et al. 2014; Kim and Nofsinger 2007; Liu et al. 2016; Namouri et al. 2018; Shi and Wang 2013).

2 See Table 4 in Appendix A for nonlinearity test results.

3 We used the “NlinTS” (Ver. 1.4.4) (Hmamouche 2020; Tank et al. 2021) package to apply the feed-forward neural network nonlinear Granger causality test in the R software.

4 We applied linear IRFs and LPIRFs by using "dplyr", "gridExtra", "ggpubr", "lpirfs" R packages. The data used in the study and relevant application codes are publicly available on github via the link: https://github.com/behaviouralfinancier/overconfidenceAI

5 Return series are logarithmic difference of the closing prices so that both variables closing price and trading volume are included in the logarithmic difference form.

6 For a calculation of the number of hidden layers/neurons, see Vujičić et al. (2016).

7 Based on AIC values, lag lengths were 2 for return and 8 for trading volume.

8 The direction of the causal relationship was only from return to trading volume. There was no statistically significant causality from trading volume to return. We reported only the results of the relevant tests because the test of overconfidence hypothesis is based on an evidence for a causality from return to trading volume. Since our main focus is finding an evidence of the presence of overconfidence and the impact of return regimes on the behaviour we did not include any exogenous variable that may also influence return or trading volume. Unreported results are available from the corresponding author on reasonable request.

9 We used logistic function to determine regimes in LPIRFs. We assumed that there was no trend. Shock type is one-unit shock. As Hodrick-Prescott filter, the value “107e9” is used for daily data, referring to the study of Lengwiler (2020).

10 Forecast horizons of impulse responses for 60 days also indicated that the response of trading volume against the return shocks persist. We did not report the results for 60 days and the results are available upon request from the corresponding author.

11 This tendency is the opposite behaviour of the disposition effect which suggests selling the winning stocks and holding the losing stocks. However, it requires further analysis with individual-level data.

12 See, Pikulina et al. (2017) and Wang (2001).

13 Wang (2001) states that nonrational investors can survive if their irrational beliefs make them trade more aggressively in the right way. Hence, the positive responses of return to the shocks from trading volume may be related to aggressive trading of overconfident investors in a right way.
